# Canine insulinoma as a model for human malignant insulinoma research: Novel perspectives for translational clinical studies

**DOI:** 10.1016/j.tranon.2021.101269

**Published:** 2021-11-15

**Authors:** Ylenia Capodanno, Barbara Altieri, Richard Elders, Annamaria Colao, Antongiulio Faggiano, Joerg Schrader

**Affiliations:** a Laboratory of Fundamental Oncology, National Cancer Center Research Institute, Chuo-ku, Tokyo 103-0045, Japan; b Division of Endocrinology and Diabetes, Department of Internal Medicine, University Hospital of Wuerzburg, Oberduerrbacher Strasse 6, Wuerzburg 97080, Germany; c London Vet Specialists, 56 Belsize Lane, London NW3 5AR, United Kingdom; d Department of Clinical Medicine and Surgery, Federico II University, Naples, Italy; e Endocrinology Unit, Department of Clinical and Molecular Medicine, Sant'Andrea Hospital, Sapienza University of Rome, Via di Grottarossa, 1035/1039, Rome 00189, Italy; f I. Department of Medicine, University Medical Center Hamburg-Eppendorf, Martinstrasse 52, Hamburg 20246, Germany

**Keywords:** Comparative oncology, Malignant insulinoma, Translational medicine, Canine model, Pancreatic neuroendocrine tumor

## Abstract

Insulinomas are considered rare indolent neuroendocrine neoplasms in human medicine, however when metastases occur no curative treatment is available thus, novel therapies are needed. Recently advances have been made in unraveling the pathophysiology of malignant insulinoma still major challenges hinder the development of a functional model to study them. Canine malignant insulinoma have similar recurrence and a poor prognosis as human malignant insulinoma. Additionally, both human and canine patients share extensively the same environment, tend to develop insulinoma seemingly spontaneously with an etiological role for hormones, at a similar incidence and stage of lifespan, with metastasis commonly to liver and regional lymph nodes, which are unresponsive to current therapies. However, the occurrence of metastases in dogs is as high as 95% compared with only 5–16% in human studies. From a comparative oncology perspective, the shared features with human insulinoma but higher incidence of metastasis in canine insulinoma suggests the latter as a model for human malignant insulinomas. With the common purpose of increasing survival rates of human and veterinary patients, in this review we are going to compare and analyze clinical, pathological and molecular aspects of canine and human insulinomas to evaluate the suitability of the canine model for future translational clinical studies.

## Highlights

• Major challenges are still faced in the treatment of human malignant insulinoma.
• None of the available insulinoma models represent faithfully the malignant disease.
• Canine insulinoma has an incidence of malignancy of 95% and a poor prognosis.
• Canine and human malignant insulinoma share clinical and molecular features.
• Canine insulinoma represents a valuable model to study the human malignant insulinoma.

## Graphical abstract

Flowchart showing how comparative oncology can improve the process of clinical trials for malignant insulinomas

**Figure:**
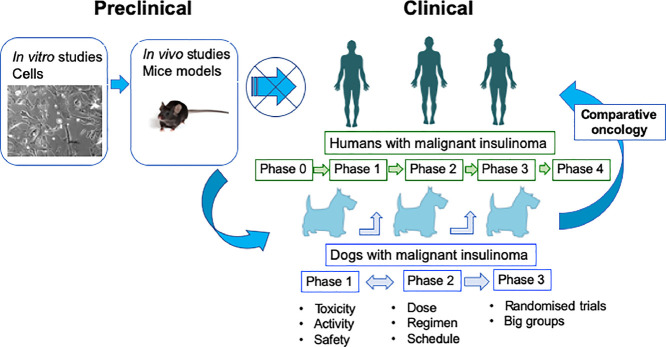

## Introduction

Insulinomas (INS) are the most common hormone-producing pancreatic neuroendocrine tumours (PNETs) [1], [2], [3]. Still, with an estimate incidence of 1–3 cases per million population per year [4], human INS are rare neoplasms. In humans, INS are often localized neoplasms readily curable by surgical resection [5]. In most cases, human INS are sporadic, solitary small lesions, with a 5-year overall survival (OS) rate of 97% [4,6]. When INS metastasize beyond the pancreas or invade the surrounding organs they are classified as malignant [3]. In 5–16% of cases, patients diagnosed with INS have lymph node and liver metastases [3]. The prolonged disease course, difficult access to pancreatic tissue during surgery, and a high tumor heterogeneity are some of the challenges of human INS treatment. Due to the low success rate of current treatment modalities for malignant INS novel targeted therapies are required [1,3,7].

To better understand the biology of INS and to identify potential new therapeutic strategies, researchers exploit on *in vitro* and *in vivo* models of INS. Specifically, the perfect model for INS would include typical insulinoma-associated mutations (*e.g. YY1*), exclude PNET mutations not associated with INS (*e.g. DAXX*/*ATRX*), retain insulin secretion and have a slow growth rate [8], [9], [10]. Unfortunately, no perfect model for the disease has been established so far.

Murine cancer models have been commonly used for underpinning the basic biology behind cancer initiation, promotion, and progression of different types of cancer [11]. However, due to differences in human and murine physiology together with the artificial conditions of mice models, they frequently cannot faithfully replicate many of the features that define cancer in humans, including long periods of latency, genomic instability, and the heterogeneity of both tumor cells and their surrounding microenvironment [12,13]. In comparison, canine cancer, as human cancer, occurs in the context of an intact immune system and often shares similar features of pathophysiology and clinical presentation to the human counterpart [11,[13], [14], [15]]. A study in 2010 in the UK showed that almost 27% of purebred dogs have died of cancer [16]. On average, cancer rate in purebred dogs is estimated to be over ten times higher than in humans. This increase in cancer susceptibility is caused by the numerous genetic bottlenecks created during the phenotypic selection of purebred traits [12,[16], [17], [18], [19]]. Interestingly, the Canine Genome Project decoded 99% of the canine genome revealing that human and canine genomes are similar enough to apply findings of one species to the other with almost ∼19,000 genes identified in the dog genome that has a similar or orthologous gene in the human genome [11].

In dogs, the incidence rate of INS has not been estimated yet, but its rate of malignancy is higher compared to humans. At the time of diagnosis approximately 95% of the cases have already developed micro-metastases [20,21]. Both human and canine patients diagnosed with malignant INS often present with an unfavorable prognosis [3,21]. The spontaneous development of INS in dogs and the similarity in clinical and biological aspects with human INS provide a rationale for the canine INS as a valuable model of the most aggressive subtypes of human INS, which are currently in most need of new therapies [20]. Thus, in this review we will first describe the currently available models for studying INS carcinogenesis and then we will focus on describing clinical, pathological and molecular similarities of INS in humans and dogs to support the appropriateness of the canine model to drive research to ultimately improve the prognosis of human and canine patients diagnosed with malignant INS.

## Current available models for studying human insulinoma

### *In vitro* models

For a few decades the cell line CM was the only available human cell line derived from an INS [22]. However throughout the years multiple questions were raised on whether this cell line represented a valuable *in vitro* model for studying human INS [23,24]. First of all, CM cell line lost insulin secretion during early passages of cell culturing. Genomic studies revealed that CM cells harbor large chromosomal re-arrangements that involve also the insulin gene, most likely causing the lack of insulin secretion in early passages [23]. Still, CM cells have not been characterized either for mutations nor for their neuroendocrine phenotype [23], [24], [25]. Even though these cells induced tumor formation in a choriollantoic membrane chicken embryo model [20], there is no publication yet describing the cell line being used for the establishment of mouse xenografts models of INS. For the aforementioned reasons, currently the CM cell line cannot be considered as a valid INS model.

In the last 20 years, a few attempts have been made to establish human INS cell lines. However, no consistent data have been presented to prove their validity as *in vitro* model for INS disease [26,27]. Only recently, a novel insulin-secreting pancreatic neuroendocrine tumor cell line called NT-3, has been established [28]. These cells retained insulin production and secretion over four years in culture (unpublished results) and human insulin secretion has been verified in a murine xenograft model of the cell line [28]. Furthermore, the cell line has a well-characterized expression profile of neuroendocrine markers resembling human beta cells [28]. Preliminary genetic analysis revealed a potentially relevant polymorphism in the *MEN1* gene (c.1621A>*G*), but failed to identify mutations in *YY1* or other typical hotspots for pancreatic neuroendocrine tumors (unpublished results). Even though the cell line might not display the typical genetic background of INS, this cell line represents the first human model of an insulin-secreting cell line derived from a metastatic INS. Still, further studies will be needed to confirm its validity as an INS *in vitro* model.

A few animal-derived *in vitro* INS model have been established in the last decades. For instance, two INS cell lines have been established from radiation induced tumors in rats, Rin5MF and INS1 [29]. Both cell lines secret insulin at various levels. Rin5MF cell line is rarely used for *in vitro* research, whereas the INS1 cell line is widely distributed. Interestingly, most of the papers published (>1000) have utilized the INS1 cells as a beta cell model rather than an INS model [30]. The phenotype and genetic background of the INS1 cells has recently been characterized. These cells have a *DAXX* and *ATRX* mutation, thus carrying typical mutations as in human pancreatic neuroendocrine tumors, but not as in INS [31]. Nevertheless, given the secretion of insulin and the slow growth rate, this cell line is up to now the best characterized *in vitro* INS model.

Additionally, a few mouse insulin-secreting cell lines are currently available including TC-6, NIT-1, MIN6 and HIT-T15 [29]. They are all derived from transgenic mice expressing the large T antigen under the control of the rat insulin promoter thus, their genetic background of p53 and Rb disruption from large T antigen expression does not resemble typical INS. Likewise, the growth rate is very high compared with typically slow-growing INS cells [29]. Thus, these cell lines qualify better for a neuroendocrine carcinoma subtype with ectopical insulin production, rather than a faithful model for human INS.

Recently, a canine insulinoma cell line, canINS, has been established [20]. This cell line has shown to cause tumor formation in a chorioallantoic membrane assay and has been characterized for the expression profile of the main neuroendocrine markers [20]. Still, insulin production is lost in early passage in adherent conditions and its mutations profile has yet to be fully characterized. Nonetheless, the expression and secretion of insulin in modified culture conditions (eg. non-adherent spheres) hold promises for using this cell line as an INS model in the future [20].

Still, so far INS cell lines have been only partially characterized and, hence, several issues still remain open regarding their neuroendocrine origin, their genomic and mutational characteristics and especially the identity of their normal counterparts from which they were originally derived. Thus, further studies are still required to develop efficient *in vitro* model for investigating INS disease.

### *In vivo* models

Besides xenografts from the INS1 and NT-3 cell lines, currently there are only two mouse models that show INS development. The first model, the RipTag mouse, shows fast growing and early fatal neuroendocrine neoplasia, which are more alike to neuroendocrine carcinomas. Nevertheless, the tumors secrete high levels of insulin producing a hypoglycemia phenotype resembling INS [32]. The second model includes the pancreatic specific *Men1* knockout model. Mouse models with conditional homozygous knockouts of *Men1* have been generated using standard Cre-Lox strategies from various promoters to target different pancreatic cell compartments. One of the most commonly used model includes mice carrying floxed *Men1* alleles (*Men1* f/f) that have been crossbred with mice expressing the Cre-recombinase from rat insulin promoter (Rip-Cre) to selectively inactivate both copies of endogenous *Men1* in the islet β-cells. In the following model pure INS develop with a long latency of up to 16 months [33,34]. Usually, in this model tumors are characterized by multiple lesions in the pancreas with dysregulated insulin production [35]. Given the typical genetic background (*e.g. MEN1* loss), the secretion of insulin, the slow growth rate and the rarity of metastases, the *MEN1* knockdown model should be regarded as the best available *in vivo* model for insulinoma.

## Comparison of canine and human insulinoma

### Incidence and risk factors

**Fig. 1:**
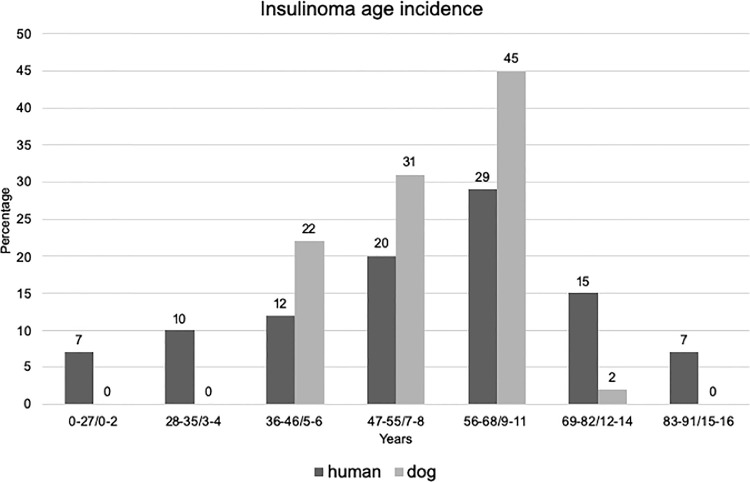
Comparisons of age incidence of insulinoma in human and dogs. Data collected from [1,3,8,38,43,46,66]. Age conversion from human to dog years was defined according to the guidelines from [98] referring to medium-size dogs.

In humans, most INS occur sporadically (90–95%% of cases), whereas 5–10% of cases are related to genetic syndrome, mainly to Multiple Endocrine Neoplasia type 1 (MEN-1). Sporadic INS have a peak of incidence from the third to the fifth decades (Fig. 1), whereas INS associated to genetic syndrome are usually diagnosed at younger age (median age less to 25 years old) [5,36,37]. INS are slightly more frequent in female patients [4].

In dogs INS usually occur sporadically with no sex predilection [38], [39], [40], [41]. Medium to large breeds including Labrador retrievers, Golden retrievers, German shepherds, Irish setters and Boxer seems to be the most common breeds diagnosed with INS [40,42]. Similarly, in dogs, the incidence is higher in middle aged dogs (9 ± 2.2 years)(Fig. 1).

### Clinical and histopathological similarities

**Fig. 2:**
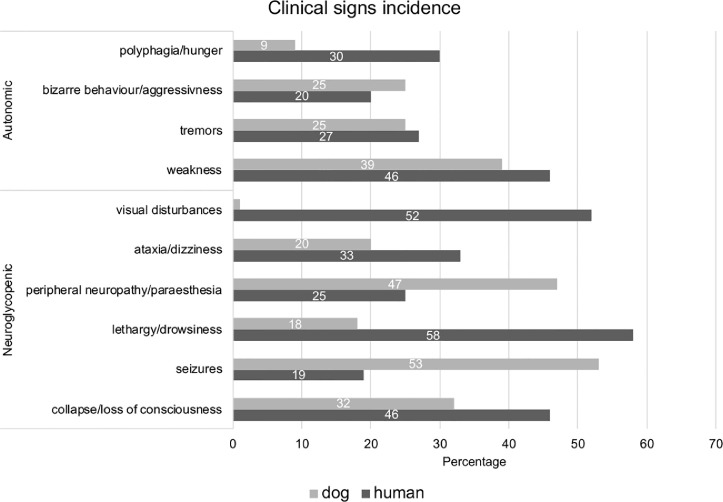
Comparison of incidence percentage of clinical signs of insulinoma in human and dogs. Data collected from [1,3,8,38,43,45,46,66].

When comparing clinical signs of INS we observe major similarities between human and canine patients (Fig. 2). In both species, symptoms/clinical signs are quite “non-specific” and do not necessarily strongly indicate an INS diagnosis until hypoglycemia is identified as the pathophysiology underlying the symptoms/clinical signs [5,7,[43], [44], [45]]. Because of the primary dependence of the brain on the metabolism of glucose for energy, most clinical signs are related to the central (Neuroglycopenic symptoms) and autonomic central nervous system (Autonomic symptoms) (Fig. 2). The most commonly occurring clinical signs such as weakness, collapse/loss of consciousness and tremors have similar frequency in human and dogs (Fig. 2). Nonetheless, seizures are more frequent in dogs than humans (53% in dogs and 19% in humans) as opposed to lethargy/drowsiness (18% in dogs and 58% in humans), perhaps as drowsiness/lethargy in dogs can be mistaken by the owners for aging and is initially regarded as insufficiently worrying for medical examination. Similarly, visual disturbances occur quite commonly in humans compared to dogs (52% in humans and 1% in dogs) [5,7,[43], [44], [45]], perhaps as visual impairment can be quite challenging to diagnose in dogs. Finally, permanent neurologic damage can develop with coma, unresponsive to glucose administration, and eventual death of canine and human patients [5,7,[43], [44], [45]].

**Fig. 3:**
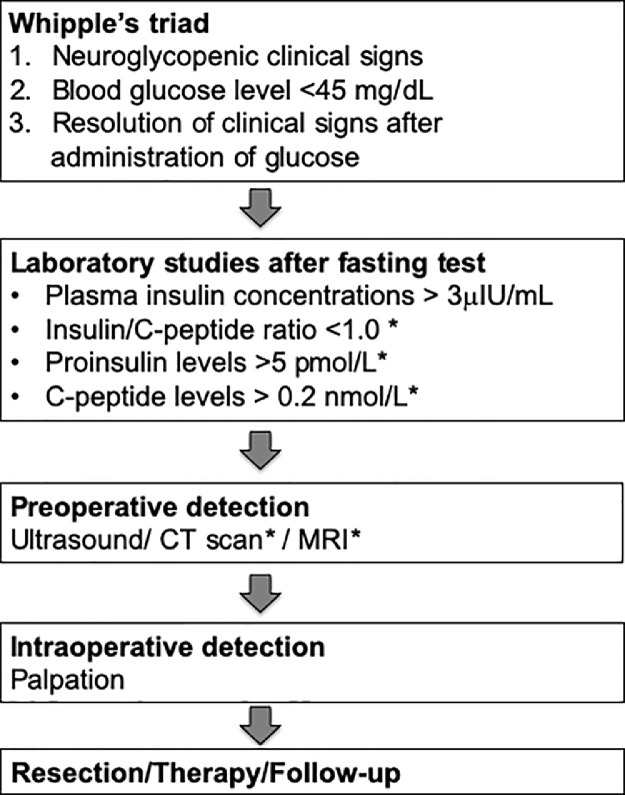
Flowchart outlining the diagnosis and the subsequent clinical tests for the detection of insulinomas in human and dogs.*indicates diagnostic tests optional in dogs. Data collected from [1,40,43].

The lack of specificity of clinical signs makes the diagnosis of INS quite difficult particularly at an early stage in both humans and dogs [1,46]. Efficient diagnosis and treatment of INS is a stepwise process (Fig. 3). The diagnosis of INS is reached through the combination of concomitant hyperinsulinemia and hypoglycemia with the exclusion of alternative diagnoses such as exogenous insulin administration [1,7,43,46,47]. In both humans and dogs, diagnosis of INS was previously obtained by documenting Whipple's triad (Fig. 3); however, it is now evident that many other disorders could respond similarly. For this reason, additional tests are often needed to confirm the diagnosis of INS-induced hypoglycaemia (Fig. 3) [42,46,48,49]. Imaging should be used as a complementary tool for diagnosing INS. In particular, imaging is usually used for preoperative localization of INS [47,50,51].

For the differential diagnosis it is important to evaluate the duration and progression of signs, moreover INS patients could have a history of seizures associated with fasting periods and exercise both in humans and in dogs [7,[38], [39], [40],^52^,^53^]. Differential diagnoses for INS include other causes of hypoglycaemia that can be broadly classified into three main groups: (i) diseases associated with excess secretion of insulin or insulin-like factors, in which excessive production of insulin can be related to islet hyperplasia or extra-pancreatic paraneoplastic syndromes; (ii) diseases where the metabolism of glucose is altered, such as adrenal insufficiency, hepatic insufficiency, glycogen storage diseases, and polycythemia; (iii) iatrogenic insulin over-dose and toxic causes of insulin release like high dose of beta-blockers [7,38,40,42,[54], [55], [56], [57]](Table 1).

**Table 1:** Differential diagnosis associated with different causes of hypoglycaemia in humans and dogs.

Causes of hypoglycemia	Human [7,[54], [55], [56]]	Dog [38,40,42,57]
**Excessive insulin production**	Endogenous hyperinsulinemic hypoglycemia (congenital hyperinsulinism, islet cell hyperplasia associated with bariatric surgery)	Islet hyperplasia
**Excessive IGF production**	Extra-pancreatic tumours (including leiomyosarcoma and liver tumors)	Extra-pancreatic tumours ((including leiomyosarcoma and liver tumors)
**Altered metabolism of glucose**	Liver disease	Liver disease
	Hypoadrenocorticism	Hypoadrenocorticism
	Hypothyroidism (severe)	
	Congenital enzyme deficiencies (glycogen storage disease type Ia and type III)	Congenital enzyme deficiencies (glycogen storage disease type Ia and type III)
	Growth hormone and corticotropin deficiency	Growth hormone deficiency
	Fanconi syndrome (renal loss of glucose)	Hunting dog hypoglycemia
**Drug-induced**	Excessive insulin administration	Excessive insulin administration
	Excessive sulfonylurea administration and pentamidine-induced hypoglycemia	Excessive administration of sulfonylurea, xylitol, aspirin, or beta-blockers
**Factitious**	Laboratory artefacts (incorrect anticoagulant/delayed separation of serum)	Laboratory artefacts (incorrect anticoagulant/delayed separation of serum)
**Systemic disease**	Severe polycythemia	Severe polycythemia
	Malnutrition	Malnutrition

Definitive diagnosis of INS can be obtained only with histopathology, therefore, before proceeding with treatment, a complete histological assessment of the tumor is usually required. However anaplastic features are often mild or inconsistent in both human and dogs making challenging to predict the biologic behavior of INS [58,59]. For this reason, the TNM staging and grading has been rearranged and adapted for this disease in humans [60] and dogs [21]. Immunohistochemically, INS of both humans and dogs stains positively for insulin, pro-insulin, chromogranin A, synaptophysin, neuronspecific enolase, cytokeratin and Ki-67 [1,3,61]. A recent study revealed that, similarly to human low-grade PNET, canine INS showed SSTR2 membranous expression, potentially supporting treatment with somatostatin analogues. Additionally, a lack of p53 nuclear staining was detected in canine INS similar to human low-grade PNETs, indicating that also in canine INS mutations of p53 rarely occur [62]. In both human and dogs, the presence of metastases, mainly located in the liver, represent the main/only definitive feature that characterizes individual tumours as malignant [3,21]. In humans a worse prognosis is associated with high Ki-67 and high serum insulin concentration at the time of diagnosis and advanced TNM stage [1]. Similarly, in dogs high Ki-67, high pre-operative serum insulin concentration, low post-operative glucose concentration, large tumor size, and advanced TNM stage indicate shorter overall survival [58].

For the detection of the INS localization both morphological and functional imaging are used. In humans, computer tomography (CT), magnetic resonance imaging (MRI) and endoscopic ultrasound (EUS) are the most used and showed a sensitivity of 47%, 58% and 89%, respectively, for the diagnosis of INS [2]. Abdominal CT is often considered the first-line imaging modality to visualize pancreatic lesions and also metastases due to its wide availability. However, MRI has higher sensitivity and specificity than CT allowing detection of small tumors (<2 cm diameters) and it currently represents the most widely used imaging method for detection of liver metastases [7]. If an insulinoma is strongly suspected but not revealed by the aforementioned modalities, additional functional imaging methods include somatostatin receptor imaging [Octreoscan® or ^68^Ga-DOTATOC/^68^Ga-DOTATATE/^68^Ga-DOTANOC positron emission tomography (PET)/CT] and glucagon-like peptide-1 (GLP-1) imaging [^68^Ga-DOTA-exendin-4 PET/CT]. Particularly, glucagon-like peptide 1 receptor (GLP-1R) imaging is a very sensitive, non-invasive method to localize benign sporadic INS. In contrast, malignant INS often lacks GLP-1Rs and overexpresses the somatostatin type 2 receptor [63]. Therefore, in case of malignant INS somatostatin receptor imaging methods are preferentially used not only for the detection of the primary tumors, but also for the detection of distant metastases [63,64]. Finally, when non-invasive imaging modalities also fail to reveal an insulinoma when highly suspected, selective arterial calcium stimulation (SACST) with hepatic venous sampling is performed. Pre-operative SACST has shown a sensitivity of >90% in identifying occult INS. However, due to its invasive nature currently SACST is used in less than 20% of human patients undergoing surgical management for INS [65]. In veterinary medicine, ultrasound (US) represents the most common tool used to visualize masses in the pancreas, as well as to detect metastatic lesions. Nonetheless, the sensitivity of US in detecting INS in dogs is reported to range from 28% to 75% and be highly operator-dependent [50]. The use of CT, MRI and functional imaging is currently limited in veterinary medicine due to the high-cost and requirement for specialized equipment, as well as the need for patient anesthesia [40,50].

### Treatment and prognosis

The curative treatment for both human and canine INS is surgery. Depending on its location, INS enucleation is the preferred surgical procedure, but partial or distal pancreatectomy or a pancreato-duodenectomy might be required. The prognosis for human and canine patients with benign INS after successful surgical resection is very favourable. Conversely, regardless of the miscellaneous therapeutic modalities for patients with malignant INS, prognosis is still poor [[1], [2], [3],^21^,^42^,^66^,^67^].

According to recent studies, in humans, the median overall survival (OS) recorded for patients with malignant INS can vary considerably depending on the presence of metastases at the time of diagnosis (range 40–143 months), with 5-and 10-year OS observed between 58 62% and 49–55%, respectively [1,3]. Occasionally, malignant INS can be surgically cured. In such cases a 5-year OS of 84% has been recorded [3]. However, surgical resection often does not represent the elective treatment for malignant INS. When surgical tumor resection is not possible, medical anti-hormonal and anti-tumor treatment are necessary. Together with changes in diet and diazoxide, which are used also before the surgery, different somatostatin analogues (SSA), including octreotide and pasireotide, have been effectively used for treating hypoglycaemic episodes [68], [69], [70]. Still, for non-surgical candidates undergoing only medical therapy the 5-years OS remains at 14% [3]. Aggressive multimodal therapy with a combination of different chemotherapeutics reagents including streptozocin, 5-fluorouracil and doxorubicin has shown to improve 5 years OS years at 24% [71]. Additionally, data from multiple clinical studies using targeted therapy against multiple steps in the IGF-R1–activated PI3K/Akt/mTOR pathway, multitargeted tyrosine kinase inhibitor, such as sunitinib and everolimus, and peptide receptor radiotherapy (PRRT), reported an improved prognosis and control of glucose level especially in malignant INS [1,44,53,[71], [72], [73], [74], [75]]. However, drugs-related adverse effects have been recorded thus, no curative treatment protocol has been yet designed for non-surgical candidates diagnosed with malignant INS [44].

In dogs, the median survival time in patients with malignant disease is 6–20 months after surgery [40,43,45,46]. When surgery is not performed the survival time is approximately of 2–8 months [38,39,45,57]. As in humans INS, in non-surgical candidates medical treatment become crucial to palliate clinical signs and for the preoperative control of blood glucose levels [39,41,46]. The medical management of canine INS is usually based on frequent small meals at least every 4–8 hr with high levels of proteins, fats, and complex carbohydrates. In conjunction with diet and exercise modifications, adjuvant therapy with diazoxide and glucocorticoids is needed both pre-operatively and in most post-operative cases to maintain euglycemia [40,42,43]. The role of chemotherapy, such as streptozocin, is not well researched in dogs [38], [39], [40], but there is emerging evidence for the use toracenib, a structurally similar drug to sunitinib, for treating canine neuroendocrine tumors [76].

### Molecular and biological similarities

In humans, the current literature suggests that INS differ from other PNET subtypes based on their clinical behavior and low *MEN1* mutation frequency [8,77]. Recent studies have documented cases of malignant, metastatic INS diagnosed years after an initial diagnosis of benign INS [78], [79], [80]. In many cases the different clinical and pathological features of benign and malignant INS suggest distinct origins of these tumors [1,[81], [82], [83]]. On this note, it has been recently hypothesized by Yu et al. that malignant INS could be derived in most cases from non-functional PNETs [84]. Countering this, multiple series have described the occurrence of secondary insulin production in a previously diagnosed non-functional PNET as a rare event, developing mostly in patients at an advanced stage of disease [1,82,83,[85], [86], [87], [88]]. Considering that most malignant INS are diagnosed when already metastatic after a long period of latency, it is possible that initially low functional activity/insulin production from tumor cells only becomes clinically significant at later stages of the disease with a higher tumor burden, causing early misdiagnosis and/or delay the accurate diagnosis of malignant INS [1,87,88]. However, direct evidence, such as lineage tracing experiments in mice, to investigate either hypothesis is currently lacking.

At a genomic level, previous sequencing studies have revealed distinctive mutational profiles when comparing INS and non-functional PNETs [8,77,89]. For instance, mutations of *MEN1, DAXX/ATRX* and the mTOR pathway genes occur in approximately 35%–65% of patients with non-functional PNETs, influencing their prognosis [8,77,90]. Whereas, less than 10% of sporadic INS are related to MEN1 syndrome, and *MEN1* inactivation by mutation only plays a minor role in tumorigenesis [20,[91], [92], [93]]. Similarly, mutations of *DAXX/ATRX* and mTOR pathway genes have rarely been observed in INS [8] although, a recent study provided evidence that an alternative lengthening of telomere phenotype (ALT) related to *DAXX/ATRX* mutations might be involved in the progression of malignant INS [94]. Recent integrated analyses of whole-genome sequencing/whole exome sequencing data have demonstrated distinctive copy-number variation and single-nucleotide variant patterns in INS and non-functional PNETs [8]. For instance, at a single-nucleotide variant level, it was revealed that approximately 30% of human INS have detectable mutations of *YY1*, reported to be the drivers of human INS tumorigenesis, while, no mutations in *YY1* were detected in non-functional PNETs [8]. In isolation, neither silencing *MEN1* nor overexpressing mutant or wild-type *YY1* induced proliferation, perhaps reflecting a requirement for additional mitogenic events, or a longer lead time to induce the requisite epigenetic changes for INS tumorigenesis [8,92].

The current literature suggests that the origin of malignant INS has yet to be fully understood with multiple events occuring at the molecular level during INS tumorigenesis [1,82,83,[85], [86], [87], [88]]. Considering that pancreatic islet cells have an inherent capability of hormonal plasticity, it would be interesting to consider in future comparative studies a scenario where clonal evolution can influence both hormone secretion and INS tumorigenesis. For instance, as secondary hormone secretion seems to be associated with disease progression as well as increased morbidity and mortality [87,88], future studies might reveal a unique malignant PNET phenotype distinct from both functional and non-functional PNETs, where delayed detectable hormone secretion could serve as a marker of tumor behavior.

In dogs, PNETs have been described mainly as functional and currently there is an absence of evidence of non-functional PNETs arising in this species, potentially due to the challenges faced in diagnosing these tumors [62]. At the genomic level, similarly to humans, canine INS usually occur sporadically and mutations of *MEN1* are not involved in the development of malignant INS [40]. A recent study revealed the transcriptomic landscape of INS in dogs [21]. For instance, it was observed that normal canine pancreas and early-stage canine primary INS have similar genetic profiles, whereas late-stage canine primary INS resembled the genetic profile of canine INS-metastatic lymph nodes. These findings suggest that in canine INS markers of malignant behavior could be identified at the primary site of the disease and that early stage/low-grade INS might have a distinct gene expression pattern compared to late stage/high-grade INS [21]. These data are consistent with what previously shown in primary and metastatic lesions of non-functional human PNETs [95], colorectal [96] and breast cancer [97]. Given that INS metastases are not easy to detect before or during surgery, these findings could help to identify those primary INS lesions with a high risk of metastasis based on their genomic features.

We compared the findings from two recent studies on the transcriptome analysis of canine INS [21] and human INS [92]. We observed multiple common biologic keys between human and canine INS identified by the DEG-enriched modules including “developmental pathways”, “insulin secretion” and “SMAD-binding”. Specifically, canonical beta-cell transcription factors such as *PDX1, NKX6.1, PAX4*, were significantly altered in both human and canine INS [21,92]. These data support beta-cell expansion and the dysregulation of the glucose-related insulin response might be central to the loss of normal glucose/insulin homeostasis forming the basis of the hyperinsulinism/hypoglycemia characteristic of INS lesions in both species. Additionally, the canine INS study cited above reported active collagen metabolism, extracellular matrix remodeling, beta-cell differentiation and non-beta-cell trans-differentiation might cause disease progression and hyperinsulinism [21]. Similarly, Wang *et al*. highlighted in human INS within the key dysregulated biologies, ‘‘extracellular matrix’’, ‘‘vasculature development’’, ‘‘cell proliferation’’, ‘‘RNA splicing’’ and ‘‘ubiquitination’’[92]. These canine and human INS data reveal a complex but often similar transformation of INS cells during carcinogenesis, while conserving insulin secretion.

Finally, recent findings using both human and canine INS cell lines, have identified a common druggable target for chemotherapy-resistant cells, the Notch pathway. Specifically, it was demonstrated that inhibiting the Notch pathway can decrease resistance to 5-Fluorouracil chemotherapy in both human and canine INS both *in vitro* and *in vivo* [20].

Taken together these data demonstrate that canine INS might share strong molecular similarities with human INS revealing novel druggable targets and the potential value of the canine model for INS clinical studies.

## Conclusions and future implications for translational studies

In summary, INS are typically indolent tumors with long latency however in malignant cases they incur a poor prognosis. Despite their apparent clinical homogeneity, INS display marked mutational heterogeneity. It appears inescapable that mutations in single genes such as *YY1* alone cannot cause INS; instead, “hits” in multiple genes are likely required which may contribute to the malignant INS phenotype. Several proteins have been suggested to stimulate tumor growth although their roles in tumorigenesis remain elusive. Thus, novel models to study the disease are required. So far, even though murine models have been useful to understand the basic mechanisms of cancer biology they cannot reproduce the complex biology of cancer recurrence and metastasis, and therefore, it is not possible to evaluate the outcomes in human patients and for the cancer drug development [13]. Considering the long and expensive process often required for drug development and the continuous failure of drugs to pass clinical trials, a quest for new solutions is needed. Comparative oncology aims to study spontaneously occurring tumours in dogs to provide relevant models for human cancer research [13,15]. Considering the clinical and molecular similarities here listed we suggest the canine INS as novel model for studying human malignant INS carcinogenesis. Thanks to an increased understanding of the molecular pathogenesis of INS, treatment approaches could be planned based on the specific behavior of these tumours and the canine model could be a crucial part of these novel achievements. Considering as well that clinical trials in pet dogs are often less restrained to the strictness of the different phases trial design as in humans, studies on dogs will help identify the tolerance and efficacy of new anticancer drugs. Translational drug development studies in pet dogs with cancer could be the answer to fill the gap between conventional pre-clinical models and human clinical trials for developing new treatments for malignant human INS. Thus, the field of comparative oncology could lead to important benefits in the context of personalized healthcare and an improved quality of life in both humans and their canine companions diagnosed with INS.

## CRediT authorship contribution statement

**Ylenia Capodanno:** Conceptualization, Data curation, Visualization, Writing – original draft, Writing – review & editing. **Barbara Altieri:** Conceptualization, Writing – original draft, Writing – review & editing. **Richard Elders:** Conceptualization, Supervision, Writing – original draft, Writing – review & editing. **Annamaria Colao:** Writing – original draft, Writing – review & editing. **Antongiulio Faggiano:** Writing – original draft, Writing – review & editing. **Joerg Schrader:** Conceptualization, Supervision, Writing – original draft, Writing – review & editing.

## CRediT authorship contribution statement

**Ylenia Capodanno:** Conceptualization, Data curation, Visualization, Writing – original draft, Writing – review & editing. **Barbara Altieri:** Conceptualization, Writing – original draft, Writing – review & editing. **Richard Elders:** Conceptualization, Supervision, Writing – original draft, Writing – review & editing. **Annamaria Colao:** Writing – original draft, Writing – review & editing. **Antongiulio Faggiano:** Writing – original draft, Writing – review & editing. **Joerg Schrader:** Conceptualization, Supervision, Writing – original draft, Writing – review & editing.
